# Tametomo no bui toukigami wo shirizoku zu

**DOI:** 10.3201/eid0807.020700

**Published:** 2002-07

**Authors:** Polyxeni Potter

**Affiliations:** * Centers for Disease Control and Prevention, Atlanta, Georgia, USA

**Figure:**
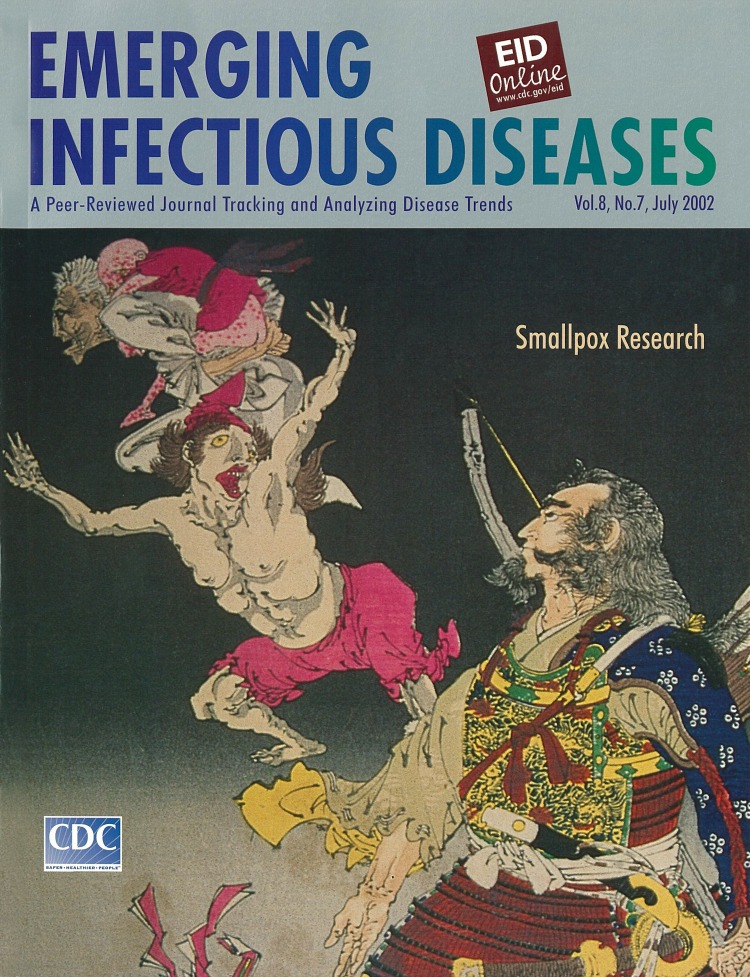
**Tametomo no bui toukigami wo shirizoku zu **Ukiyo-e woodcut print by Yoshitoshi, c. 1890 Naito Museum of Pharmaceutical Science and Industry, Hashima, Gifu, Japan

The first record of smallpox in Japan was found in the Nihon Shoki, published in 735 (the 7th year of Tempyo). The incident was also described in Ishinho, the oldest medical book in Japan, issued by Yasuyori Tanba in 984 (the 2nd year of Eikan). Smallpox, called Hoso in Japanese, came to Japan in the same era as Buddhism. The disease was considered very dangerous. Even those who recovered could have pockmarks or loss of sight. Parents were constantly concerned about their children becoming ill with smallpox.

The color red was used in prints and other smallpox illustrations because it was believed that Hoso-Kami, the god of smallpox, felt strongly about this color. When the skin rash was purple, the patient’s condition was considered serious. If the rash turned red, the patient would recover safely. Shoni-Hitsuyo-Yoikugusa, written by Gyuzan Kazuki in 1798 (the 10th year of Kansei), recommended that children with smallpox be clothed in red garments and that those caring for the sick also wear red. 

"Hoso-e" color prints against smallpox were used in prayers to boost the morale of ill children. After the patients recovered, these pictures were burned or floated down the river. Therefore, few examples are left of prints in which the color red predominates. The pictures drawn as protection against smallpox depicted heroic figures to give people courage against smallpox. Tametomo, a heroic samurai, was a representative genie. Legend has it that Tametomo was once banished to Hachijyo Jima, a small island far from main island in Japan, and that is why smallpox never occurred there.

Suggested citation: Potter P. Tametomo no bui toukigami wo shirizoku zu [about the cover]. Emerg Infect Dis [serial on the Internet]. 2002 Jul [date cited]. Available from: http://www.cdc.gov/ncidod/EID/vol8no7/v8n7cover.htm
